# Adult Wilms’ Tumour: Case Report and Review of Literature

**DOI:** 10.15586/jkcvhl.2016.52

**Published:** 2016-05-23

**Authors:** Sunny Modi, Kor Woi Tiang, Po Inglis, Stuart Collins

**Affiliations:** 1 Urology Department, Greenslopes Private Hospital, Brisbane, Australia; 2 Royal Brisbane and Women’s Hospital, Brisbane, Australia; 3 Sunshine Coast University Private Hospital, Sunshine Coast, Australia

**Keywords:** adult Wilms’ tumour, nephroblastoma, renal tumour

## Abstract

Wilms’ tumour (nephroblastoma) is the most common renal tumour in children. Wilms’ tumour in adults is extremely rare and has a poorer prognosis than paediatric Wilms’ tumour. It is difficult to differentiate adult Wilms’ tumour from renal cell carcinoma based on radiological findings alone. The diagnosis in adults is often serendipitous following nephrectomy for presumed renal cell carcinoma. Because of the paucity of literature, there are no standard protocols for the management of adult Wilms’ tumour, and therefore, it is managed as per paediatric Wilms’ tumour. Herein, we report the case of adult Wilms’ tumour in a 43-year-old man, which was diagnosed unexpectedly following nephrectomy for presumed renal cell carcinoma.

## Introduction

Wilms’ tumour, named after the 19^th^ century German surgeon Carl Max Wilhelm Wilms, is probably derived from primitive metanephric blastema (1). Wilms’ tumour, also known as nephroblastoma, is the most common paediatric cancer of the kidney, occurring mainly in the first 5years of life with peak incidence between 3 and 4 years of age (2). Wilms’ tumour is very rare in adults with an incidence of about 0.2 per million per year (3) and represents less than 1% of all diagnosed renal tumours (4). There are approximately 300 documented cases of adult Wilms’ tumour in the literature to date (5). Symptomatology of adult Wilms’ tumour differs significantly compared with that of children’s. Adults normally present with abdominal pain and haematuria while in children, the tumour is frequently a painless, rapidly enlarging abdominal mass that is often readily palpable. Because of its anatomical position, venous drainage and lymphatic drainage, it is not uncommon for distant metastasis to the lungs and liver. Metastasis to the bone, skin, bladder, large intestine, central nervous system and the contralateral kidney is rare (3).

There is no significant histological and radiological difference between adult and childhood Wilms’ tumour (2). In fact, they share the classical triphasic histopathological features such as blastemal, epithelial and stromal features under the microscope. The histological appearance is characterized by marked structural diversity, and the occurrence of all three types in the same case is uncommon (1). Adult Wilms’ tumour is diagnosed based on well-known criteria described by Kilton et al. (6). They include the following: 1, the tumour under consideration should be a primary renal neoplasm; 2, presence of primitive blastemic spindle or round cell component; 3, formation of abortive or embryonal tubules or glomerular structures; 4, no area of tumour diagnostic of renal cell carcinoma; 5, pictorial confirmation of histology and 6, patient’s age should be >15 years. In 1980, Kilton et al. (6) reported 35 cases of adult Wilms’ tumour complying with all the above criteria.

Clinical outcome for adults is inferior when compared with children, although better results are reported when treated within paediatric trials (7). Multiple factors including the unfamiliarity of oncologists and pathologists with adult Wilms’ tumour, lack of standardized treatment, and consequent delays in initiating the appropriate risk-adapted therapy may contribute to the poor outcome. Because of the fact that nephroblastoma is a very rare type of cancer, adult patients are treated based on the available treatment protocols used for children, which was developed by the National Wilms’ Tumour Study Group (NWTS) in North America, and the International Society of Pediatric Oncology (SIOP) (8).

## Case report

**Figure 1.**
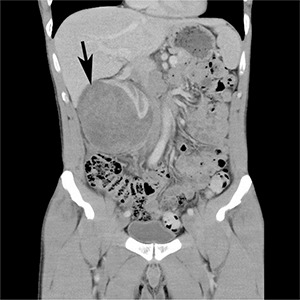
CT scan – coronal view. Large mass lesion (arrow) within right kidney measuring up to 7.4 × 7.5 × 9.2 cm.

**Figure 2.**
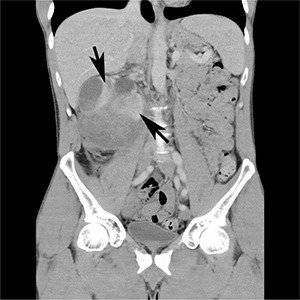
CT scan – coronal view showing right kidney mass with subcapsular haematoma (arrows).

A 43-year-old Caucasian man, with a 4-week history of right upper quadrant and right flank pain radiating to his right testicle, presented to his general practitioner. There was no preceding trauma, haematuria or any other systemic symptoms. The past medical history included osteoid osteoma in the left upper femur, which was removed in 2007. He also had L4/5 microdiscectomy. There was no positive family history of medical problems or cancer. He was a non-smoker, had no allergies and was not taking any regular medications. The general practitioner organized an ultrasound, which revealed a large subcapsular haematoma measuring 7 × 5 × 7 cm in the right kidney compressing the renal cortex. Further investigation with computed tomography (CT) scan of the abdomen with intravenous contrast revealed enlarged right kidney with subcapsular haematoma and a mass lesion within the right lower pole measuring up to 7.4 × 7.5 × 9.2 cm with associated paracaval lymphadenopathy producing compression of the right renal vein and inferior vena cava (**Figures 1 and 2**). The patient was then referred to an urologist for further management.

Physical examination revealed that the right kidney area was filled, with minimal tenderness. The haemoglobin concentration was 140 g/L. There was a mild elevation of creatinine (116 µmol/L), and the estimated glomerular filtration rate was 66 ml/min. CT staging showed a large right renal tumour and significant paracaval lymphadenopathy producing compression of the right renal vein and inferior vena cava. The size of the tumour was substantially increased in size to 11.5 × 11.6 × 11.5 cm when compared with that in the previous CT scan.

**Figure 3.**
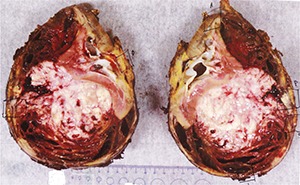
Right kidney specimen – cream and pink colour tumour with mixture of solid and cystic components with haematoma in the lumen of the cyst and possible necrosis.

**Figure 4.**
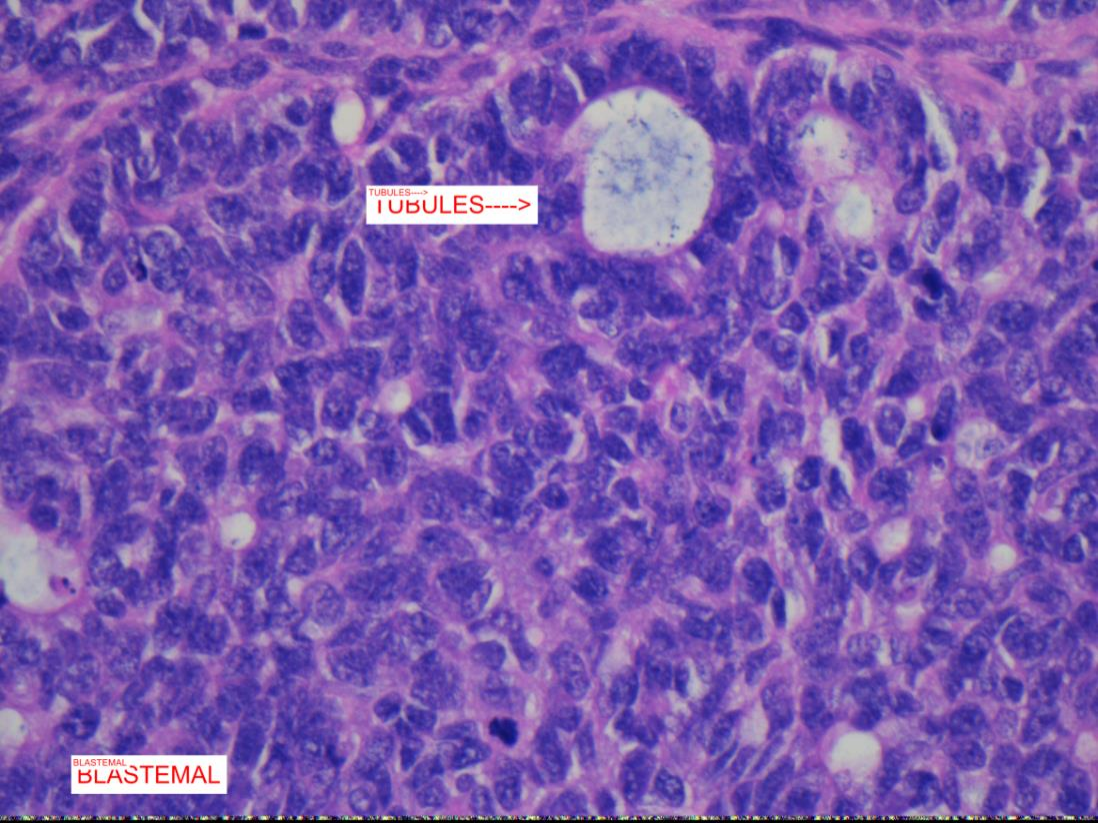
Microscopic features of adult Wilms’ tumour showing blastemal elements.

**Figure 5.**
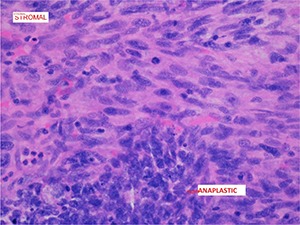
Microscopic features of adult Wilms’ tumour showing stromal elements and focal anaplastic elements.

A preliminary diagnosis of renal cell carcinoma was made based on the clinical findings and investigations. The patient underwent open radical right nephrectomy, retroperitoneal lymph node dissection and partial excision, and reconstruction of vena cava. The renal samples were sent for histopathology. The right kidney specimen (with fibro adipose tissue) weighed 1291 g and 175 × 140 × 110 mm in size. The tumour was located in the mid-pole and lower pole of approximately 100 × 95 × 100 mm. On gross inspection, the tumour was cream and pink in colour with mixture of solid and cystic components (**Figure 3**). There was an extensive haematoma within the lumen of the cyst and possible necrosis. The retrocaval lymph node measured 4 cm. Histologically, the tumour comprised of blastemal, stromal and anaplastic elements. The blastemal elements were present in nodular fashion and had syncytial pattern with no diffuse infiltrative component (**Figure 4**). The anaplastic elements were focally present in stromal elements (nuclear sized 3× tumour cells and tripolar mitosis; **Figure 5**). The stromal elements were present with focal anaplastic elements. There were features of necrosis, haemorrhage and cystic degeneration. The morphologic and histopathology features favoured adult Wilms’ tumour with synovial sarcoma as a differential diagnosis. A fluorescence in situ hybridization analysis showed loss of heterogeneity (LOH) for 1p 16q, which is associated with poor outcomes in paediatric Wilms’ tumour. The pathological staging was pT3a, pN1 and cM0 (pT3a – tumour invading renal sinus fat but not beyond Gerota’s fascia, pN1 – metastasis in single regional lymph node, cM0 – not stated). The patient was subsequently treated with adjuvant combination chemotherapy and radiotherapy.

Based on the age of presentation, unfavourable histology and chromosomal LOH for 1p16q, the tumour was considered high-risk, and chemotherapy was initiated as per the Children’s Oncology Group UH-1 protocol, including early radiation to the tumour bed, and multiagent chemotherapy including actinomycin D, vincristine and doxorubicin. Radiation and chemotherapy were commenced concurrently within 3 weeks of surgery. The intensity of chemotherapy was maintained as per protocol with granulocyte support. The patient has been followed up at regular intervals with CT scans and routine blood tests. There has been no radiological or clinical recurrence at the end of 12 months of initial diagnosis.

## Discussion

Wilms’ tumour is the most common renal tumour accounting for 85% of paediatric renal neoplasms. The tumour is most prevalent in the first 5 years of life with peak incidence between 3 and 4 years of age (2). The annual incidence rate in children under 15 years is 7 to 10 cases per million, accounting for 6–7% of all paediatric malignant tumours (9). The spontaneous rupture and haemorrhage as presenting signs of adult Wilms’ tumour also are very rare (8). It is difficult to diagnose adult Wilms’ tumour preoperatively because there are no specific radiographic findings (6).

Treatment of Wilms’ tumour invariably is multimodal involving surgery, chemotherapy and/or radiotherapy. Two accepted management protocols are the NWTS and SIOP. NWTS has always recommended upfront nephrectomy to define the accurate stage of the tumour and the histology, on which further treatment stratification is decided. In contrast, the SIOP investigators pioneered the concept of prenephrectomy chemotherapy in all patients over 6 months of age to reduce the tumour size and prevent intraoperative tumour rupture causing spillage and to increase the proportion of children with a lower tumour stage that required less overall treatment (10).

The NWTS group has reported an overall survival rate of 82% in adults with favourable histology Wilms’ tumour (11). Terenziani et al. (5) reported their institutional experience regarding adult Wilms’ tumour wherein 17 patients with adult Wilms’ tumour who were older than 16 years were treated according to paediatric Wilms’ tumour guidelines. In this series, the overall survival was 62.4% at 5 years. Reinhard et al. (12) reported their experience with 30 cases of adult Wilms’ tumour. A complete remission was achieved in 24 patients. Event-free survival was 57%, and the overall survival was 83%. They concluded that adults can be cured in a high percentage by a multimodal treatment according to paediatric protocols.

It is well documented that Wilms’ tumours are radio- and chemo-sensitive, and delay in treatment can adversely affect outcomes (4). There are a number of case series that suggest that the outcome for adult Wilms’ tumour has improved when paediatric treatment approaches, including multimodality chemo- and radiotherapy adapted from the paediatric treatment protocols, are used (4, 11–13). Long-term follow-up of patients not only concerns the risk of relapse but the cardiotoxicity, neurotoxicity, hepatotoxicity and nephrotoxicity of the chemotherapeutics.

## Conclusion

Adult Wilms’ tumour is a rare entity with mainly case reports-only literature. There are no established protocols for treatment of this group of patients. Being so rare, the diagnosis is generally made on postoperative pathology. Treatment of this tumour is multimodal incorporating surgery and lengthy chemotherapy, which needs to be tailored to the patient. Its prognosis is poorer than that of Wilms’ tumour in the paediatric population. Relapse in the first year after treatment is a predictor of poor outcome. It is recognized that Wilms’ tumour in adults is a rare entity, and the importance of registering such patients to international data bases will assist in the research and development of future management guidelines for this tumour.
